# A Dynamic Model for Strategies and Dynamics of Plant Water-Potential Regulation Under Drought Conditions

**DOI:** 10.3389/fpls.2020.00373

**Published:** 2020-04-28

**Authors:** Phillip Papastefanou, Christian S. Zang, Thomas A. M. Pugh, Daijun Liu, Thorsten E. E. Grams, Thomas Hickler, Anja Rammig

**Affiliations:** ^1^TUM School of Life Sciences Weihenstephan, Technical University of Munich, Freising, Germany; ^2^School of Geography, Earth and Environmental Sciences, University of Birmingham, Birmingham, United Kingdom; ^3^Birmingham Institute of Forest Research, University of Birmingham, Birmingham, United Kingdom; ^4^Senckenberg Biodiversity and Climate Research Centre, Frankfurt, Germany; ^5^Department of Physical Geography, Goethe University, Frankfurt, Germany

**Keywords:** climate change, plant-hydraulics, leaf water potential, isohydricity, drought, water stress

## Abstract

Vegetation responds to drought through a complex interplay of plant hydraulic mechanisms, posing challenges for model development and parameterization. We present a mathematical model that describes the dynamics of leaf water-potential over time while considering different strategies by which plant species regulate their water-potentials. The model has two parameters: the parameter *λ* describing the adjustment of the leaf water potential to changes in soil water potential, and the parameter Δψ_ww_ describing the typical ‘well-watered’ leaf water potentials at non-stressed (near-zero) levels of soil water potential. Our model was tested and calibrated on 110 time-series datasets containing the leaf- and soil water potentials of 66 species under drought and non-drought conditions. Our model successfully reproduces the measured leaf water potentials over time based on three different regulation strategies under drought. We found that three parameter sets derived from the measurement data reproduced the dynamics of 53% of an drought dataset, and 52% of a control dataset [root mean square error (RMSE) < 0.5 MPa)]. We conclude that, instead of quantifying water-potential-regulation of different plant species by complex modeling approaches, a small set of parameters may be sufficient to describe the water potential regulation behavior for large-scale modeling. Thus, our approach paves the way for a parsimonious representation of the full spectrum of plant hydraulic responses to drought in dynamic vegetation models.

## Introduction

Droughts are projected to increase in frequency and intensity within the 21st century (e.g., Dai, 2013; IPCC, 2014) and are expected to have severe effects on whole ecosystems, e.g., inducing large-scale tree mortality and vegetation die-off (Anderegg et al., 2012; Rowland et al., 2015; Choat et al., 2018). Quantifying these effects and their impact on ecosystem function requires robust modeling approaches, which capture the key features of how plants respond to drought. This challenge is particularly acute for estimating plant responses to drought using large-scale dynamic vegetation models (DVMs, e.g., Cramer et al., 2001) that would need to account for a variety of different vegetation types with different hydraulic systems and strategies of responding to drought. One way to classify those strategies is using the concept of isohydricity (Jones and Sutherland, 1991; Tardieu and Simonneau, 1998), which assumes that plant water potential is strongly coupled to stomatal behavior. The classical isohydricity concept differentiates between isohydric plants, which limit stomatal conductance as soil water potential decreases, thereby approaching constant leaf water potentials, and anisohydric plants, which keep their stomata open, continuing photosynthesis and transpiration. Recently, it has been shown that these two strategies form a continuum rather than a dichotomy (Klein, 2014). Furthermore, it seems that regulation of leaf water-potential and stomatal control, the two mechanisms that are assumed to be strongly connected in the original definition of isohydricity, are less interdependent than originally thought (Martínez-Vilalta and Garcia-Forner, 2017) and a range of metrics to assess isohydricity have been proposed (Feng et al., 2019). Nonetheless, it is clear that understanding the likely response of ecosystems to drought will require modeling approaches that explicitly consider the range of possible hydraulic strategies plants can adopt.

Existing hydraulic model frameworks describe the interplay among the leaf- and soil water potentials, stomatal conductance and transpiration under both well-watered and drought conditions. These models implement detailed hydraulic states, such as leaf water potential, stomatal behavior and xylem conductivity (Jones and Sutherland, 1991; Leuning et al., 1995; Tardieu et al., 2015; Mirfenderesgi et al., 2016; Sperry et al., 2016, 2017; Venturas et al., 2018). Many hydraulic models are based on optimization principles to predict stomatal conductance based on hydraulic properties (Mencuccini et al., 2019), but differ in their optimization criteria and conditions. For example, Dewar et al. (2018) maximized carbon gain, whereas Mackay et al. (2015); Sperry et al. (2017), and Venturas et al. (2018) maximized the difference between carbon gains and hydraulic risk. Other approaches control the stimulus responses on physiological or molecular scales (Tardieu et al., 2015). Parameterization of these models beyond the site-scale remains, however, a challenge. For example, Sperry et al. (2016) need to cover a large space of parameter values in their model to account for the observed heterogeneity in hydraulic behaviors. Additionally, the hydraulic model frameworks described above are mostly stand-alone models and often ignore the coupling to other ecosystem processes, which would be necessary for the implementation of ecosystem responses to drought in DVMs.

Dynamic vegetation models have been developed to simulate the impacts of climate change on ecosystem processes, such as photosynthesis, carbon uptake and allocation, growth, competition and mortality, and the interplay between carbon-, water- and (more recently) nutrient cycles, at large scales (Cramer et al., 2001; Smith et al., 2014; Achat et al., 2016). These models usually describe the plant hydraulics by very simple formulations, and only a few have implemented a more detailed hydraulic representation (e.g., Hickler et al., 2006; Xu et al., 2016; Eller et al., 2018; Kennedy et al., 2019). Capturing variation in strategies for leaf water potential regulation has, however, remained a challenge for two reasons: (1) existing hydraulic models are parameterized for specific sites for a given range of environmental drivers, but dynamic vegetation models need to represent a larger scale across which the environmental drivers often differ widely and (2) the parameters of hydraulic models, which define different plant strategies, are not available for a wide range of species or plant functional types needed for large-scale simulations. A high amount of poorly constrained parameters would induce substantial uncertainty in simulations. Thus, the challenge for large-scale modeling is to develop a parsimonious representation of hydraulic behavior (e.g., leaf water potential dynamics), which can capture a range of different strategies of responding to drought, with parameterization still being feasible.

The aims of our study are thus (1) to provide an empirical hydraulic framework that explicitly captures the regulation mechanisms of water potential in different plant species over time, (2) to reproduce observed leaf water potential dynamics, and (3) to provide a generalized set of parameters that reproduces the dynamics across datasets and species. We present a new framework, based on differential equations, which dynamically describes water-potential regulation mechanisms in plants and can be used for implementation in large-scale dynamic vegetation models. It builds on a static isohydricity classification framework developed by Martínez-Vilalta et al. (2014) but goes beyond their approach by considering leaf water potential regulation dynamically over time. We provide parameter estimates for different hydraulic strategies for the parameterization of plant functional types, as commonly used in dynamic vegetation models.

## Materials and Methods

### Model Description

#### Principles of Plant Water Flow

Water flows from the roots through the stem to the leaves, where it is released to the atmosphere. Based on these general principles, our model assumes that the water flow is driven by a forcing pressure Δψ(*t*) that changes over time:

(1) $$\Delta \,\psi \,\left(t\right)=\psi_{s}\,\left(t\right)-\psi_{\text{L}}\,\left(t\right)-\rho \,g\,h$$

where, Δψ_*s*_(*t*) and ψ_L_(*t*) are the changes in soil and leaf water potential over time, respectively. The gravitational pull is given by ρ⋅*g*⋅*h*, where ρ is the sapwood density, *g* is the gravitational acceleration and *h* is the canopy height (Table 2). Equation 1 assumes no contributions from plant water storage to Δψ(*t*).

#### Modeling the Forcing Potential Under Well-Watered Conditions and Isohydricity

In the absence of water stress, a plant experiences a daily average forcing pressure Δψ, which we here denote as the forcing potential under well-watered conditions Δψ_ww_:

(2) $$\Delta \,\psi_{\text{ww}}=\frac{1}{T}\,\int_{0}^{T}\Delta \,\psi \,\left(t\right)\,dt\approx c\,o\,n\,s\,t.$$

where *T* = 24 *h*.

Physiologically, the calculation of Δψ_ww_ is motivated by the isohydrodynamic behavior of plants, which ensures a nearly constant Δψ over time (Domec and Johnson, 2012). Similarly, Martínez-Vilalta et al. (2014) introduced the so-called ‘pull parameter’Λ, which accounts for the pulling capacity of the plant under high (near-zero) soil water potential. In this situation, abundant water is available in the soil. Our approach implicitly assumes that leaf water potential, and thus, stomatal opening (Supplementary Methods S1), responds to changes in soil water potential, either via hydraulic or chemical signals (Larcher, 2003; Kuromori et al., 2018; Yoshida and Fernie, 2018) or by directly affecting the leaf water potential ψ_L_. In the following, we formalize three special cases of isohydricity (Roman et al., 2015), and present a general solution encompassing also the full continuum in between the cases.

##### Case 1: Extreme isohydric behavior (Figure 1)

**FIGURE 1:**
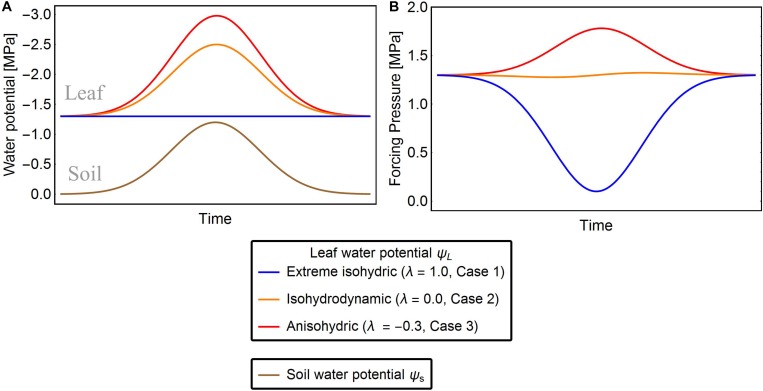
Exemplary solutions of solutions of Eqn. 7 showing ψ_L_ **(A)** and Δψ **(B)** of the three strategies of water-potential regulation for given ψ_*s*_. The red, orange, and blue lines are the leaf water potentials for *λ* = –0.3, 0.0, and 1.0, respectively. In extreme isohydric regulation, a decline in soil water potential does not affect the leaf water potential, but the forcing pressure falls and recovers as the soil water potential drops and rises, respectively (blue lines). In isohydrodynamic regulation, the leaf water potential follows the soil water potential, while the forcing pressure remains constant (orange lines). In anisohydric regulation, the leaf water potential is decreasing and the forcing pressure is increasing with declining soil water potential (red lines).

Leaf water potential ψ_L_ in this case is assumed to be constant (Franco, 1998), thus, the change of ψ_L_(*t*) with respect to $\psi_{s}\text{is}\mathrm{zero}\left(\frac{d\,\psi_{\text{L}}}{d\,\psi_{s}}=0\right)$ and Δψ decreases as the soil dries (Figure 1, blue line). Here we set ψ(*t*) to its minimal value ψ_L,min_ which is actively maintained by the plant: ψ_L_(*t*)→ψ_L,*min*_. This tendency of ψ_L_ can be expressed as a differential equation with an adjustment rate *r* that accounts for response lags:

(3) $$\frac{d\,\psi_{\text{L}}}{d\,t}=-r\,\left(\psi_{\text{L}}\,\left(t\right)-\psi_{L,\min}\right)$$

Assuming that Δψ_ww_ is reached when the soil water is abundant [i.e., ψ_*s*_(*t*) approximates zero], we can relate ψ_L,min_ to Δψ_ww_ as follows:Δψ_ww_ = ψ_*s*_(*t*)−ψ_L,*min*_≈−ψ_L,*min*_. The differential equation is then refactored as

(4) $$\frac{d\,\psi_{\text{L}}}{d\,t}=-r\,\left(\psi_{\text{L}}\,\left(t\right)-\left(-\Delta \,\psi_{\text{ww}}\right)\right)=-r\,\left(\psi_{\text{L}}\,\left(t\right)+\Delta \,\psi_{\text{ww}}\right)$$

##### Case 2: Isohydrodynamic behavior

In Case 2, plants are assumed to adjust their leaf water potential ψ_L_ to follow changes in $\psi_{s}\left(\frac{d\,\psi_{\text{L}}}{d\,\psi_{s}}=1\right)$ maintaining a constant forcing pressure Δψ (Figure 1, orange line) and thereby also their maximal stomatal opening. Under constant forcing Δψ→Δψ_ww_ (Franks et al., 2007), the rate of change in ψ_L_ is defined as

(5) $$\frac{d\,\psi_{\text{L}}}{d\,t}=r\,\left(\Delta \,\psi \,\left(t\right)-\Delta \,\psi_{\text{ww}}\right)$$

The isohydrodynamic behavior described by Eq. (5) adjusts ψ_L_ in two ways: (1) If Δψ(*t*) is lower than the maximum Δψ_ww_, it increases until it reaches Δψ_ww_; (2) If Δψ(*t*) is greater than Δψ_ww_, it decreases towards Δψ_ww_.

##### Case 3: Anisohydric behavior

Under drought stress, anisohydric plants may adjust their ψ_L_ until Δψ increases $\left(\frac{d\,\psi_{\text{L}}}{d\,\psi_{s}}>1\right)$, hence plants exhibiting anisohydric behavior can potentially adapt their leaf water potentials so that Δψ exceeds Δψ_ww_ (Figure 1, red line) (Roman et al., 2015). To model this situation, we assume that the leaf water potential tends to *M*:=*q*⋅Δψ_ww_ with *q* > 1, and refactor Eq. 5 as

(6) $$\frac{d\,\psi_{\text{L}}}{d\,t}=r\,\left(\Delta \,\psi \,\left(t\right)-M\right)=r\,\left(\Delta \,\psi \,\left(t\right)-q\cdot \Delta \,\psi_{\text{ww}}\right)$$

Finally, we present a general solution for all three cases, introducing an isohydricity factor λ that captures the transition between the three cases:

(7) $$\frac{d\,\psi_{\text{L}}}{d\,t}=r\,\left(\left(\left(1-\lambda \right)\,\psi_{s}-\psi_{\text{L}}\,\left(t\right)\right)-\Delta \,\psi_{\text{ww}}\right)$$

Here, λ measures the isohydricity or hydraulic behavior of the water potential regulation. When λ = 1, Eq. (7) reduces to Eq. (4) describing case 1, when λ = 0 it becomes Eq. (5) describing case 2, and when λ < 0 (case 3) we can define $q:=\frac{\Delta \,\psi_{\text{ww}}+\lambda \,\psi_{s}}{\Delta \,\psi_{\text{ww}}}$ by Eq. (7). Assuming that leaf water potential adjusts rapidly on the daily time scale of our study, we set $r=\frac{1}{d\,a\,y}$. Note that our modeling approach based on Eq. (7) can simulate the full spectrum of different hydraulic behaviors, including the three special cases described above. Similar frameworks were proposed by Martínez-Vilalta et al. (2014), whose *σ* measure is inversely related to λ, and by Sperry et al., 2016, who distinguished between isohydricity and anisohydricity by their *Slope* parameter (which also inversely scales with our λ). However, in contrast to the approach of Martínez-Vilalta et al. (2014), which is static, our model is able to reproduce the dynamics (temporal changes) of ψ_L_ across different ψ_*s*_ conditions. Sperry et al., 2016 also capture temporal, diurnal changes in leaf water potential, but require considerably more parameters. Recent studies point toward a non-linear relation between ψ_L_ and ψ_*s*_ (Meinzer et al., 2016), which is also considered in our approach, when using small parameter *r* (i.e., on a sub-daily time scale).

### Observational Data

We synthesized publications listed in Martínez-Vilalta et al. (2014) including the measured predawn water potentials (ψ_PD_), the midday water potentials (ψ_MD_) and plant height *h* (for calculating the gravitational pull) for woody plants. We extracted time series of ψ_PD_ and ψ_MD_ from each of the publications (Supplementary Data Sheet S1) using WebPlotDigitizer (Rohatgi, 2018). We selected only those datasets containing at least four measurements of ψ_PD_ and ψ_MD_ over a timespan of at least 2 weeks. Because very few experiments reported the sub-daily measurements of ψ_L_ across multiple days, such data were omitted from our analysis. Additionally, we included the dataset from Roman et al. (2015), which already contained the time series of measured ψ_PD_ and ψ_MD_ in electronic format.

We used ψ_PD_ and ψ_MD_ as proxies of ψ_*s*_ and ψ_L_, respectively. These assumptions hold if ψ_*s*_ equilibrates overnight with the wettest soil layers around the active roots (Martínez-Vilalta et al., 2014). Under severe drought conditions, overnight equilibration can be insufficient and ψ_*s*_ can exceed ψ_PD_ (Xu et al., 2016). The opposite situation (ψ_*s*_ < ψ_PD_) is also possible because root xylem embolism and root shrinkage can create air spaces around the roots, thereby preventing equilibration (Cuneo et al., 2016). These extreme cases, which will violate our proxy assumptions, are excluded from the general parameterization of our model.

**FIGURE 2:**
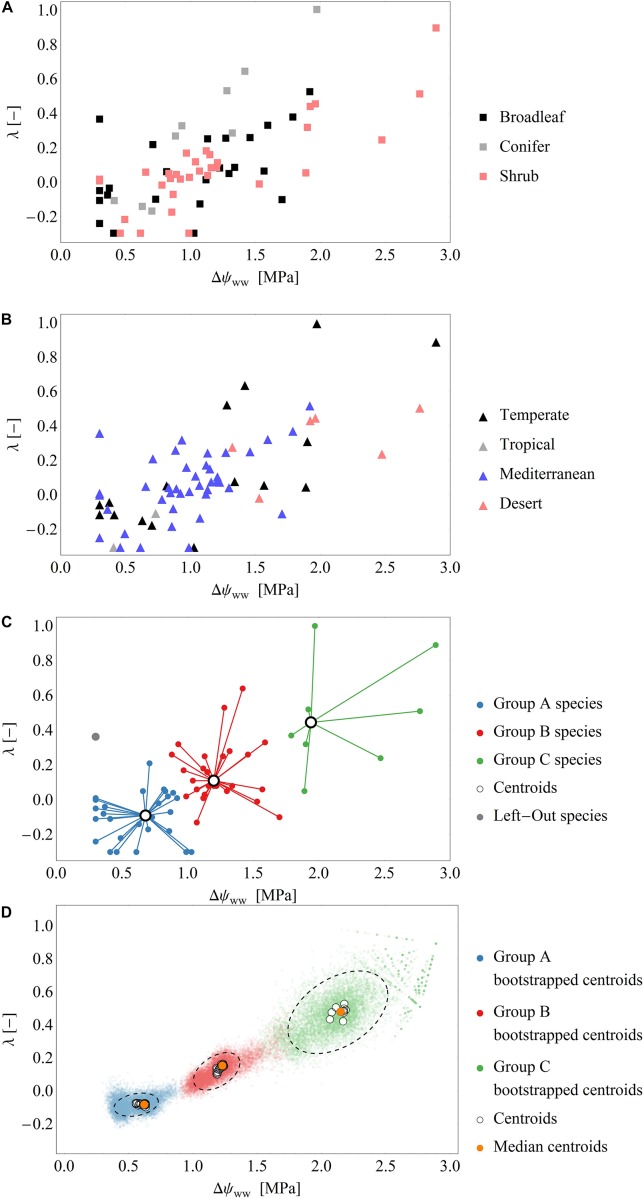
**(A,B)** Scatterplots of the fitted parameter pairs across the drought dataset, grouped by functional types **(A)** and biomes **(B)**. **(C)** Exemplary classification of fitted parameters into the three groups by the cluster analysis using the leave-one-out approach (see also Supplementary Figure S1). Of the 66 data points in the drought dataset 65 points (blue, red, green) are used for clustering and one point (gray) is used for model evaluation. Black open circles are the cluster centroids of the three groups. **(D)** Results over all clustering analysis performed by the leave-one-out analysis: Blue, green, red dots: Centroids of an additional bootstrapped clustering analysis performed. Black, dashed ellipsis: 90%-quantile-ellipses encompassing 90% of the bootstrapped centroids. Black open circles are the cluster centroids of the three groups of the 66 clustering analysis. Orange dots: Median centroids based on the centroids of each cluster analysis (open circles).

In total, we derived 110 time series of 66 species across temperate, tropical, Mediterranean and desert biomes. To sub-divide our dataset, we distinguished between non-drought (‘control’) and drought conditions. Those time series exhibiting pronounced changes in ψ_PD_ (varying by at least 1 MPa across the measuring period) were assigned to drought periods. After this division, we derived 66 time series of 48 species for the drought dataset and 44 time series of 44 species for the control dataset. Fourteen species were represented in both, drought and control datasets. The measurements under drought conditions consisted of broadleaved (*n* = 28) and coniferous tree species (*n* = 8), shrubs (*n* = 31). The control measurements consisted mainly of broadleaved (*n* = 41) and coniferous tree species (*n* = 9), but also included shrubs (*n* = 4) (Figure 2A). The data collected under drought conditions were mainly (∼90%) collected from temperate zones (*n* = 20) and the Mediterranean (*n* = 42), while only few data were available from deserts (*n* = 6) and tropical areas (*n* = 2) (Figure 2B).

### Model Calibration: Estimation of the Parameter Pairs Δψ_ww_ and λ

The model parameters were calibrated by fitting Eq. (7) to the drought dataset. Using the measured ψ_PD_ time series as ψ_*s*_ in Eq. (7), we optimized the parameter pair (Δψ_ww_, λ) by minimizing the root mean square error (RMSE) between the observed ψ_L_ (measured ψ_MD_) and predicted ψ_L_. We also investigated the stability of the parameter sets by computing a relative root mean square error $R\,R\,M\,S\,E\,\left(\Delta \,\psi_{\text{ww}},\lambda \right)=\frac{R\,M\,S\,E\,\left(\Delta \,\psi_{\text{ww}},\lambda \right)}{R\,M\,S\,E\,\left(\Delta \,\psi_{\text{ww}},\hat{\lambda }\right)}-1$ with $\left(\hat{\Delta \,\psi_{\text{ww}}},\hat{\lambda }\right)$ being the parameter pair where $R\,M\,S\,E\,\left(\hat{\Delta \,\psi_{\text{ww}}},\hat{\lambda }\right)$ is a global minimum across the parameter space. To ensure convergence toward a global minimum parameter set, we employed three different heuristic numerical optimization techniques (‘random search,’ ‘simulated annealing’ and ‘differential evolution’; see Supplementary Methods S2). The numerical optimization routines were restricted to certain ranges of λ and Δψ_ww_. In particular, *λ* was limited to λ < 1.0 (Case 1, perfect isohydric behavior) and to at least λ > −0.3 (Case 3, anisohydricity) to scale linearly with cases 1 and 2. Δψ_ww_ was maintained above 0.3 MPa to allow a minimal positive forcing difference between the leaf- and soil water potentials, which was visible in all of our datasets. These settings are rationalized in the ‘stability analysis’ section below.

### Clustering/Grouping of Parameter Pairs and Model Evaluation

We classified the observations in terms of water-potential regulation mechanisms based on a *k*-means clustering analysis (Python-Package: *sklearn*) of the model parameters (Δψ_ww_, λ). Because of the small size of our dataset we selected the leave-one-out cross validation technique (Efron, 1982) to test the robustness of our approach. Thereby, all points except one from our drought dataset are used to identify three clustering groups and one (left out) point is used for model evaluation. The *k*-means cluster analysis was also used to predict the corresponding group of the left out point and centroids of the three clusters. This clustering analysis was iterated over all points of the drought dataset resulting in 66 different clustering analyses with three groups, three centroids and one left out point, each.

We calculated the median centroids for each of the three groups over all the clustering analyses. Each median centroid is again a parameter pair (Δψ_ww_,λ).

Each left out point was associated with one of the three clustering groups using the *k*-means cluster analysis. By applying the groups centroid parameter pair, we compared ψ_L,*pred*_(*t*) against the time series ψ_L,*obs*_(*t*) of the left out data point. As the control dataset was not used for model calibration and hence no group was associated with it, we applied the three median centroids to each point of the control dataset.

**FIGURE 3:**
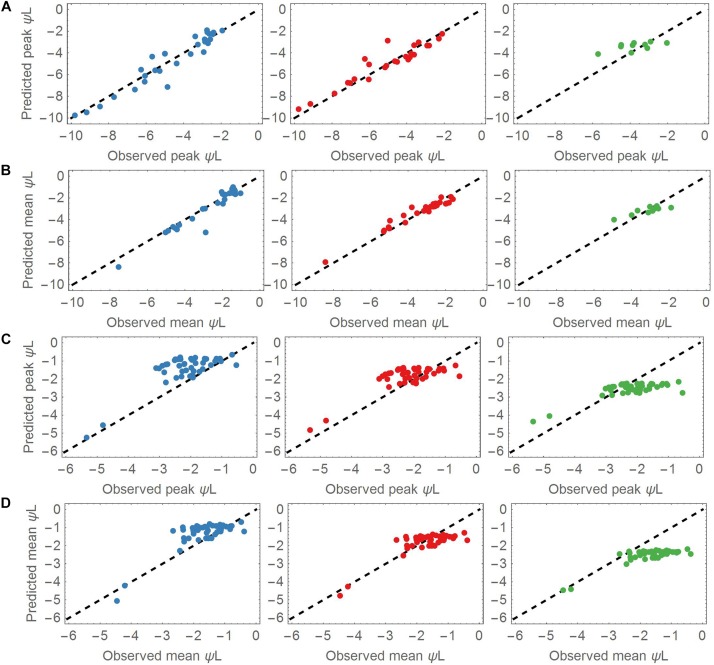
**(A)** Predicted versus observed peak leaf water potentials in the drought dataset for the three cluster groups. Most of the species are covered by the groups A (Blue points, left column) and B (Red points, middle column). A lower number of species was associated with group C (Green points, right column). **(B)** As for panel **(A)**, but plotting the mean values of the predicted and observed leaf water potentials. **(C)** Predicted versus observed peak leaf water potentials applied to all control datasets. The observed peaks aggregate around –2 MPa. **(D)** As for panel **(C)**, but applying the mean values of the predicted and observed leaf water potentials to the control datasets. The observed means also aggregate around –2 MPa.

**FIGURE 4:**
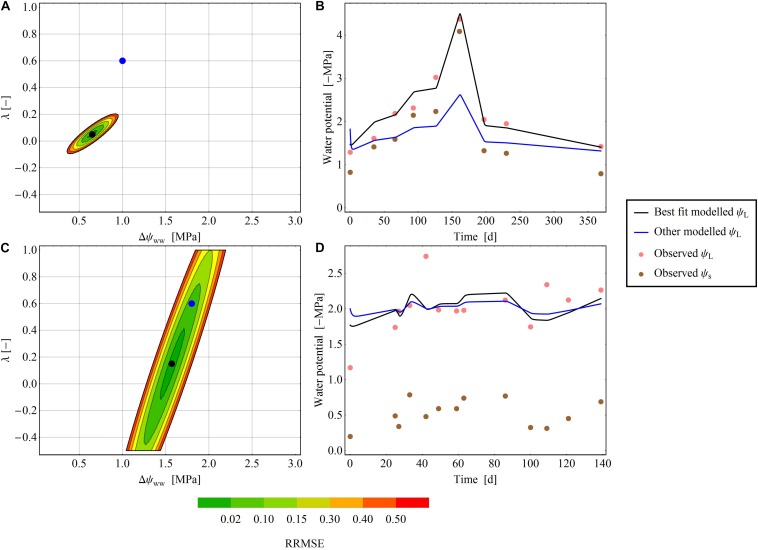
Areas of RMSE deviation from the optimal parameter pair (Δψ_ww_ and λ) for two exemplary species at two exemplary sites. **(A,B)** Pratt et al. (2008) dataset, where an exact parameter pair is readily determined. **(C,D)** Ellsworth and Reich (1992) dataset: an exact determination of the parameter pair is not possible. **(A,C)** The color gradient shows the RRMSE [from 0 (green) to 0.2 (red)] between the selected and minimal RMSEs. In the white areas of the plot, the RRMSE exceeds 0.2. The black dot denotes the optimal parameter pair in the dataset, and the blue dot is another parameter pair that diverges from the best pair. **(B,D)** Show the time-series of the modelled ψ_L_ for the best fit (black line) and the other fit (blue line) next to observed ψ_L_ (pink points) and observed ψ_*s*_ (brown points).

**FIGURE 5:**
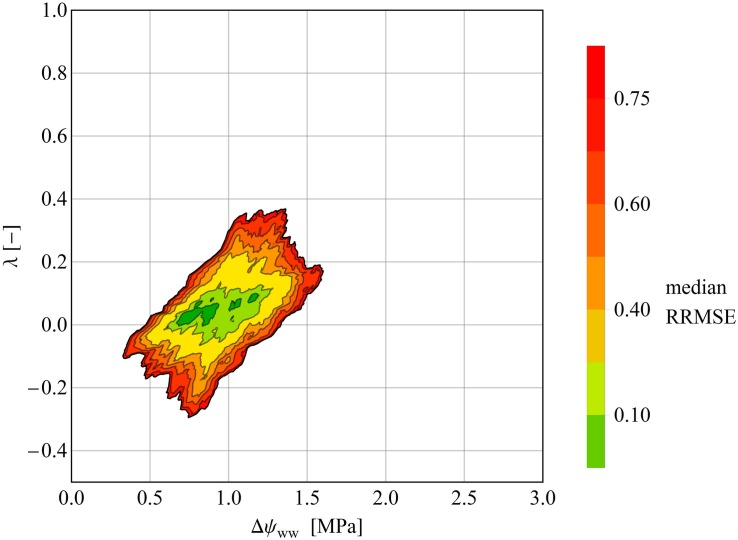
Median RRMSEs at each pixel in the (Δψ_ww__ww_–, λ) parameter plane, computed over all species of the drought dataset. All values above 3.0 MPa were excluded. Like the clustering analysis, the isohydrodynamic water-potential regulation (Case 2) minimizes the error across the drought datasets. The Δψ_max_– and *λ*-axes are each subdivided into 1000 parameter values, giving (1000 × 1000) = 1000.000 pixels in the parameter plane.

The overall prediction skill was evaluated by calculating the RMSE of the observed versus the modeled leaf water potential. Additionally, we evaluated the dynamic behavior of the model, and compared the mean and peak (minimum) leaf water potentials of each of the three groups centroid and the corresponding left out point (Figure 3). Determining the peak leaf water potential under drought stress is reasonable, because the risk of stem (or branch) cavitation is thought to be highest at this potential. Equation (7) was then solved (1) using the associated group’s centroid Δψ_ww_ and λ values for the drought dataset and (2) using the median centroids of the groups for the control dataset. Next, the time point *t*_P_ obs_ of the observed peak ψ_L_ was identified in each time series, and compared with the predicted peak ψ_L_ at *t*_P_ sim_. In a given time series, the means and peak values of all observed ψ_L_ values were compared against the predicted mean and peak ψ_L_ values. The evaluation measure was the RRMSE (Figures 4, 5).

## Results

### Estimated Parameter Pairs

After fitting the model (i.e., Eq. 7) to the drought dataset, we obtained a broad range of both parameters Δψ_ww_ and λ (mean Δψ_ww_ = 1.1 MPa, *SD* = 0.59 MPa; range of Δψ_ww_ 0.3 to 2.89 MPa and mean λ = 0.1, *SD* = 0.27; range of λ−0.3 to 1.0, Figures 2A–C). Generally, ∼90% of the time series were best fitted when λ < 0.5, and 33% were best fitted when λ < 0.0. These results indicate a tendency toward isohydrodynamic (Case 2) or anisohydric (Case 3) water-potential regulation of the examined species (Figures 1, 2). In the Δψ_ww_ fitting, 78 and 27% of the estimated values were lower than 1.5 and 0.75 MPa, respectively.

After grouping the parameter pairs by biomes and functional types, there were no obvious clusters of specific water-potential regulation groups (Figure 2B). The mean λ was 0.06 (*SD* = 0.19) for the Mediterranean species, 0.17 (*SD* = 0.39) for the temperate species, and 0.32 (*SD* = 0.19) for the desert species. The mean Δψ_ww_ was high for the desert species (2.0 MPa) and lower for the temperate and Mediterranean species (1.18 MPa and 0.97 MPa, respectively). This suggests that (relative to the ψ_*s*_) the ψ_L_ is lower in desert species than in species occupying other habitats. There is some evidence that desert species generally maximize photosynthesis rates rather than minimizing transpiration (e.g., Gibson 1998) which could explain the higher Δψ_ww_. The mean λ were similar in broadleaved trees and shrubs (λ = 0.08, *SD* = 0.25), but higher in conifers (λ = 0.29, *SD* = 0.39), indicating a more isohydric water-potential regulation).

The mean Δψ_ww_ values were similar across broadleaved trees, shrubs and conifers (1.0, 1.18, and 1.06 MPa, respectively). Generally, grouping the parameters by biome or functional type did not show a clear separation of the two model parameters. This indicates that in our dataset, the different regulation mechanisms of hydraulic water potential were spread across biomes and functional types.

### Clustering of Parameter Pairs and Model Calibration

The cluster analysis results in the splitting of the parameter pairs into three distinguished groups (Table 1). About 44% of species in the drought dataset (29/66) were characterized by low Δψ_ww_ (mean = 0.61 MPa) and λ (mean = −0.08; group A). A second group (group B, 41% of the drought dataset 27/66) displays higher (moderate) values of Δψ_ww_ (mean = 1.22 MPa) and λ (mean = 0.15). The remaining 15% (10/66) species from group C with high λ and higher Δψ_ww_. Below we describe these three groups and their regulation behavior in more detail:

**TABLE 1:** Overview of the water-potential regulation groups obtained in the cluster analysis and their mean (Δψ_ww_, λ) values.

Group	Mean centroid Δψ_ww_	Mean centroid λ	*n*^∗^
Group A (low Δψ_ww_, λ)	0.62	−0.08	29
Group B (moderate Δψ_ww_, λ)	1.23	0.15	27
Group C (high Δψ_ww_, λ)	2.15	0.49	10

*^∗^Number of observations (parameter pairs) in each group.*

**TABLE 2:** Abbreviations, names and units of all variables and parameters used in our modeling approach.

Abbreviation	Name	Unit
λ	Isohydricity factor	–
Δψ_ww_	Well-watered (maximum) forcing pressure	MPa
ψ_L_	Leaf water potential	MPa
ψ_s_	Soil water potential	MPa
Δψ	Forcing pressure	MPa
ρ	Density of water	kg m^–3^
*g*	Gravitational pull	m s^–2^
*h*	Plant height	m
*g*_C_	Canopy conductivity	m s^–1^
*k*_s_	Sapwood conductivity	m s^–1^
*A*_S_	Sapwood area	m^2^
*A*_L_	Leaf area	m^2^
*VPD*	Vapor pressure deficit	MPa
Φ	Soil water content	m^3^ m^–3^

• Group A (low Δψ_ww_, λ): Comprising most of the parameter pairs (*n* = 29), this group was characterized by particularly low values of λ (−0.3 to 0.36, mean = −0.08, *SD* = 0.16) and low values Δψ_ww_ (0.3–1.03 MPa, mean = 0.62 MPa, *SD* = 0.25 MPa). Species in this group lie between isohydrodynamic and anisohydric water-potential regulators (Cases 2 and 3), which strongly adapt their ψ_L_ to changes in ψ_*s*_.
• Group B (moderate Δψ_ww_, λ): The second largest group (*n* = 27) was also characterized by low λ (−0.13 to 0.64, mean = 0.15, *SD* = 0.17), but higher values of Δψ_ww_ (0.88 to 1.7 MPa, mean = 1.23 MPa, *SD* = 0.21 MPa). The water potential regulation in this group settles between isohydric and isohydrodynamic (Cases 1 and 2). Group B species adopt less strongly to changes in the soil water potential compared to species of group A. Because of their higher Δψ_ww_ values compared to group A, plants of this group generally operate at lower levels of ψ_L_ (relative to ψ_*s*_) than group A species.
• Group C (high Δψ_ww_, λ): The smallest group in the clustering analysis (*n* = 10) was characterized by higher **λ** values compared to group A and B (mean = 0.48, *SD* = 0.28) and hence by a more isohydric water-potential regulation. The higher **Δψ_ww_** values in this group (mean = 2.15 MPa, *SD* = 0.4 MPa) imply also operation at lower levels of ψ_L_ (relative to ψ_*s*_).

About 44% (29 out of 66) entries in the drought dataset were assigned to the low-Δψ_ww_, *λ* group (group A), indicating that a large part of our dataset could be described by an isohydrodynamic water-potential regulation parameterization of our model (Cases 2 and 3). Species in this group strongly adjust their ψ_L_ after a drop in ψ_*s*_. This group also spans a range of low Δψ_ww_, indicating that under unstressed conditions, isohydrodynamic water-potential regulation is associated with low gradients. Almost the same amount of entries (27 out of 66) in the drought dataset were assigned to the moderate Δψ_ww_ group (group B). In contrast to group A entries of group B had slightly higher λ and considerably higher Δψ_ww_ values. The water-potential regulation of group B species is less anisohydric compared to group A, tending more toward isohydrodynamic behavior. The higher Δψ_ww_ implies that species of this group generally operate at lower levels of ψ_L_ (relative to ψ_*s*_). A minority of species (10/66) were assigned to the high Δψ_ww_ group (group C). The high values of Δψ_ww_ indicate that species associated with this group generally operate at low levels of ψ_L_ relative to ψ_*s*_, however, are less vigorously adjusting ψ_L_ to decreases in ψ_*s*_ than the groups A and B. Species of group C can be considered as being between isohydric and isohydrodynamic, but tending more toward isohydric behavior.

### Model Evaluation of the Clustered Groups

The parameter sets of group A (low Δψ_ww_) and B (moderate Δψ_ww_) represented most of the dynamics of the drought dataset. Overall, group A and B adequately predicted the mean and peak leaf water potentials of the drought dataset (Figures 3A,B). The mean RMSE of the predicted leaf water potential in Group A was 0.51 MPa, whereas the observed and predicted peak ψ_L_ values in the drought dataset ranged from −1.93 to −9.81 MPa, <reflecting the wide range of ψ_L_ covered by this dataset and the prediction. Both the observed and predicted ψ_L_ peaks varied largely across the dataset (mean = −4.65 MPa, *SD* = 2.19 MPa). Similarly, group B accurately explained ψ_L_ time series of the 27 points associated with it (mean RMSE = 0.52 MPa). The peak ψ_L_ observed and predicted of group B covered a slightly wider range from −2.11 to −12.03 MPa with similar variability but lower peak ψ_L_ (mean = −5.56 MPa, *SD* = 2.65 MPa). With a mean RMSE = 0.63 MPa, group C captured ψ_L_ time series of its 10 associated species with less accuracy. Compared to the species of group A and B the species associated with group C had higher observed and predicted ψ_L_ ranging from −2.04 to −5.72 MPa. Its values centered around higher values of peak ψ_L_ with lower variability (mean = −3.75 MPa, *SD* = 1.02 MPa).

The mean observed ψ_L_ of group A was significantly higher than the peak observed ψ_L_ ranging from −1.03 to −7.54 MPa with mean around −2.66 MPa. Again, group B showed a similar range of mean observed ψ_L_ from −1.62 MPa to −8.43 MPa and a more negative mean of −3.37 MPa. The differences compared to peak observed ψ_L_ reflect the strong changes of ψ_L_ in the measuring period, and a tendency toward anisohydric or isohydrodynamic water-potential regulation. Group C showed mean observed ψ_L_ ranging from −1.88 to −4.92 MPa which is closer to the peak observed ψ_L_ compared to groups A and B. This indicates the more isohydric behavior of species associated with this group as they do not lower their leaf water potential under changing ψ_L_ as strong as group A and B.

The observed ψ_L_ peaks in the control datasets ranged from −0.54 to −8.92 MPa (mean observed ψ_L_ range −0.38 to −7.82 MPa), with both peaks and means clustered around −2 MPa. The smaller difference in mean and peak ψ_L_ indicates less pronounced changes of ψ_L_ within the measuring period (Figures 3C,D). These findings show that under non-drought conditions, most of the plants stabilized their ψ_L_ around −2 MPa. Group A and B best predicted the leaf water potential time series ψ_L_ in the control time series (with RMSEs of 0.58 and 0.55 MPa, respectively). Group C performed worse in predicting the ψ_L_ time series (RMSE = 1.02 MPa).

### Stability Analysis in the 2D Parameter Plane

In some time series, the RRMSE was restricted to a small area of the parameter space, indicating one parameter pair that describers the regulation mechanism of the water potential according to the parameter λ (Figure 4A). This means, that predictions using the best fitting ψ_L_ trace time series of observed ψ_L_ much more closely than alternative predictions based on different parameter pairs (Figure 4B). For other time series the RRMSEs around the best parameter pair covered a larger area, resulting in ambiguous parameterization of Δψ_ww_ and λ in the time series (Figure 4C). Here, different pairs of (Δψ_ww_, λ) can describe the observed dataset and consequently a single regulation mechanism of water potential cannot be identified (Figure 4D). After averaging the RRMSEs across the drought dataset for each pixel (Δψ_ww_, λ) in the parameter plane, the median lowest errors were determined as 0.6 MPa <Δψ_ww_<1.4 MPa, and −0.5 < λ < 0.125 (Figure 5), consistent with the median centroids of groups A and B.

## Discussion

### Strategies of Leaf Water Potential Regulation

We found the majority of species displaying more isohydrodynamic regulation of their leaf water potential (Case 2, Figure 1). In contrast, strong isohydric water potential regulation (Case 1, Figure 1) was found in only a few cases. However, we found a large variability within these hydraulic strategies, which is reflected by the large spread of our model parameter λ across species, in agreement with findings of Martínez-Vilalta et al. (2014).

When reducing the heterogeneity of our model parameter λ to only a small set of parameter pairs (Δψ_ww_, λ), we could successfully reproduce the water-potential-regulation dynamics of the considered species under both drought and control conditions. The model showed better performance in predicting the responses to drought compared to the control responses (Figure 3). This may be because our model is particularly designed to only capture drought responses to declining levels of ψ_*s*_; Under control conditions with constant ψ_*s*_ only other environmental drivers may influence ψ_L_, which are not covered by our approach.

In particular, group A and B in the cluster analysis explained a wide range of the leaf water-potential dynamics (Figure 3) across species. These two groups represent plants that isohydrodynamically regulate their water potential by maintaining a constant forcing potential Δψ (Figure 1). The differences in hydraulic behavior between the two groups A and B mainly arise (according to the clustering) from their operational levels of ψ_L_ under drought and control conditions. With a higher Δψ_ww_ species associated with group B are generally working at lower levels of ψ_L_ compared to species of group A. This indicates that species of group B might be slightly more drought resistant than species of group A.

This reduction of parameters is especially relevant for dynamic vegetation modeling, because models often assume extreme isohydric water-potential regulation in plants (e.g., Hickler et al., 2006), which rarely occurred in our dataset. Differences in hydraulic behavior such as water-potential regulation can have substantial implications for ecosystems. For instance, more anisohydric behavior may substantially influence evapotranspiration fluxes. Furthermore, above 85% loss of conductivity induced by cavitation trees experience widespread mortality (Venturas et al., 2018).

Group C comprised less species of our dataset and also its prediction skill was lower, which might be either because, the more isohydric strategies were not widely spread across species, or (more likely) because our dataset was biased toward European and North American species and Mediterranean and temperate biomes (see section Materials and Methods). Additionally, our dataset was biased toward broadleaved species. Thus, the lack of significant differences in the water potential regulations between conifer and broadleaved species may result from the low representation of conifer species. The absolute levels of soil water potentials varied across species and sites, especially in the drought- but also in the control-datasets. Thus, our approach not only captured the differences in hydraulic strategies, but also the intensity in differences between the normal and drought treatments, and the different environmental conditions, which are implicitly reflected in ψ_*s*_.

### Stability of Model Parameters Versus Input Datasets

In the stability analysis, we found large differences in the uniqueness of the model parameter pairs (Δψ_ww_, λ) across datasets, environmental conditions and species. Some datasets yielded a clearly determined parameter pair (Δψ_ww_, λ) (Figures 4A,B), with a RMSE that rapidly increased with increasing distance from the best fit pair; other datasets yielded a larger set of parameter pairs with equal RMSEs (Figures 4C,D). Generally, the fitting achieved more unequivocal parameter pairs (Δψ_ww_, λ), which could be more distinctly attributed to a water-potential regulation strategy from time series with (1) a longer measuring period, (2) a larger number of sample/measuring points, and (3) a larger variety in ψ_*s*_ over time. This indicates that the quality of a unique parameter pair (Δψ_ww_, λ), and hence the integrity of the predicted water-potential regulation strategy, depends on capturing both drought and non-drought conditions within the dataset. In control treatments with constant high ψ_*s*_ (close to zero), multiple parameter pairs (Δψ_ww_, λ) produced similar ψ_L_, so the regulation mechanism of the water potential cannot be clearly defined (Figure 4D).

### Model Limitations

Our model focuses on describing leaf water potential from changes in soil water potential assuming that leaf water-potential regulation can be modeled independently of VPD. However, VPD has a strong influence on hydraulic conductance of plants, in particular during drought, when high VPD leads to decreasing whole-plant hydraulic conductance and increasing canopy temperature (Zhang et al., 2017), indicating stomatal closure of plants to prevent water loss. Stomatal closure reduces transpiration, which is crucial for cooling leaves under high ambient air temperatures often associated with severe droughts (Teskey et al., 2015). Therefore, drought-stressed plants face a trade-off between leaf overheating bearing the risk of potential severe leaf damage, and low xylem-water potential bearing the risk of cavitation. The drought datasets applied here were measured in rainfall exclusion experiments or artificial drought experiments, often only prevent rainwater from reaching the soil (da Costa et al., 2010), but rarely account for impacts of persistent atmospheric dryness under extreme drought (Liu et al., 2015, 2018) and may thus underestimate the impacts of drought. To fully represent the ecosystem responses under drought, such feedbacks need to be considered in drought experiments and implemented in vegetation models. Here, we neglected the direct influence of VPD on the leaf water potential, mainly because time series of VPD were available only for a limited number of datasets. However, when deriving the stomatal conductance from leaf water potential, VPD cannot be excluded as a driving factor (Medlyn et al., 2011).

Another important mechanism that needs to be considered when modeling leaf water potential, is that even when stomata are closed, plants continue to loose water through the stomata and the cuticle (Duursma et al., 2019). In particular, under extreme drought conditions and consecutive days of high VPD, cuticular water loss plays an important role. This water loss reduces the xylem water potential, and may potentially cause plant death by cavitation (Choat et al., 2018). Future hydraulic models should also incorporate water loss through both, leaf cuticula and closed stomata during severe droughts (Duursma et al., 2019).

Our present study focused on the regulation of water potential in plants. Explicit formulations of the water storage capacities of stems, leaves and roots, which are incorporated in other approaches (Xu et al., 2016), were not considered, primarily because of a lack of parameterization data. Although the stem water potential and stem water capacity are correlated, their dynamics and interactions are complex and differ across species. In our model, the parameter *r* can be interpreted as a storage capacity because changes in *r* would cause temporal shifts of ψ_L_ relative to ψ_*s*_. Because none of the time series in the datasets showed a visible lag effect of ψ_L_ to changes in ψ_*s*_ we set a high value of *r* = 1 (implying lags of less than 1 day). However, the species and/or regions of the experiments may have been insensitive to capacitance. Where capacitance is known to be important, as in some tropical species (Borchert and Pockman, 2005), this assumption may need to be revisited.

### Relating the Leaf Water-Potential Regulation and Canopy Conductivity Implications for Implementations in Dynamic Vegetation Models

We provide a possibility to connect leaf water potential regulation and the forcing pressure Δψ to canopy conductivity (Supplementary Methods S1) based on the principle that the imposed transpiration flux balances the sapwood flux induced by the forcing pressure Δψ (Whitehead et al., 1984). The forcing pressure Δψ and canopy conductivity can be linked by Darcy’s law (McDowell et al., 2016). By this link, the effects of leaf water potential regulation can be used to estimate to canopy conductivity and stomatal behavior according to the three special cases: A decrease of Δψ of extreme isohydric plants (case 1, Figure 1) would lead to a decrease in canopy conductivity *g*_C_, reduced transpiration, and consequent reduction in photosynthesis and carbon uptake. Keeping Δψ constant under drying soil requires maintaining a high *g*_C_ (isohydrodynamic plants, case 2), ensuring that transpiration and photosynthesis continue under increasingly dry conditions. However in this case, xylem cavitation under severe soil-moisture stress can decrease the *g*_C_, thus lowering the xylem conductivity *k*_*s*_. Finally, anisohydric plants (case 3) adjust their ψ_L_ until Δψ actually increases (Figure 1). Plants adopting this strategy maintain a high *g*_C_ and high transpiration- and photosynthesis rates, even under strong drought stress. Loss of xylem conductivity *k*_*s*_ induced by cavitation is compensated by the decrease in ψ_L_ and increase in Δψ. For a summary of the three cases see also Supplementary Methods S3.

Changes in leaf water potential are commonly simulated by differential equations in dynamic vegetation models (Xu et al., 2016; Eller et al., 2018). However, the differences in isohydricity or the regulation mechanisms of water potential are not parametrized in these models. Our new approach explicitly accounts for the differences in water potential mechanisms among plant species by specifying two parameters (Δψ_ww_ and λ) and can be applied in dynamic vegetation models. Our model is also technically capable of simulating water potential dynamics on sub-daily timescales. However, this is not tested here, because sub-daily measurements of leaf water potential across many consecutive days are expensive and only available from very few experiments.

If the median cluster parameter pairs of our cluster groups A, B, C indeed represents a major fraction of plants, it can be generalized to many vegetation models. However, this tentative conclusion must be tested by further analysis. Furthermore, dynamic vegetation models increasingly aim to capture trait diversity and to understand its implications for ecosystem resilience (e.g., Scheiter et al., 2013; Sakschewski et al., 2016). Here, we could derive the trait gradients encapsulating different regulation strategies of leaf water potentials. Finally, to improve the accuracy of the leaf water-potential dynamics for a specific species, the model parameters can be derived if species-specific time-series of the predawn and midday leaf water potentials would be available (Supplementary Tables S1, S2).

## Conclusion

We presented a novel modeling approach that captures the temporal dynamics of regulation strategies of leaf water potentials in plants under changing soil water potentials, with strategies ranging from extreme isohydric (case 1), through isohydrodynamic (case 2), to anisohydric (case 3). With only two parameters (Δψ_ww_ and λ, assuming *r* = 1), our model captures different water potential regulation mechanisms across species accurately on a daily scale. Although we did not find a general solution for leaf water potential regulation across all species, many species’ leaf water potential regulation could be modeled using one particular parameter combination. We suggest that when implementing our framework into dynamic vegetation models, a few parameter combinations may be enough to model the leaf water potential regulation across the available plant functional types. To verify this, it is needed to test, in particular, whether the parameter λ can represent (1) a constant parameter, (2) a species-specific trait parameter or (3) a dynamic property of a species. This question arises because parameterizations of our groups A, B and C adequately represented many different datasets, species and environmental conditions.

Our results (especially the successful application of the mean parameter sets of λ and Δψ_max_) show that the isohydricity concept of water-potential regulation in our and other approaches (e.g., Martínez-Vilalta et al., 2014) cannot explain the hydraulic differences among plant species. Alongside water potential regulation, we should further investigate the different stomatal behaviors and hydraulic safety margins, and the relations among them (Anderegg et al., 2018). Overall, our parsimonious approach offers two main advantages: (1) it is easily tested against observations of leaf- and soil water potentials, and (2) it is easily implemented in dynamic vegetation models to predict leaf-water-potential over time.

## Data Availability Statement

The time-series of the dataset analyzed in this manuscript can be found in the Supporting Information. Requests for a complete list of journal articles investigated by this study should be directed to papastefanou@gmail.com.

## Author Contributions

PP and AR conceived the study and wrote the first draft of the manuscript. All authors contributed to the development of the model and to the writing of the manuscript.

## Conflict of Interest

The authors declare that the research was conducted in the absence of any commercial or financial relationships that could be construed as a potential conflict of interest.

## Supplementary Material

DATASHEET S1 Time series of predawn and midday leaf water potential.
