# Supplementary material for: A Dynamic Model for Strategies and Dynamics of Plant Water-Potential Regulation Under Drought Conditions
Source: Front Plant Sci. 2020 Apr 28;11:373. doi: 10.3389/fpls.2020.00373 (PMC7199548; doi:10.3389/fpls.2020.00373)
Supplement: Supplementary file 2 [file Presentation_1.pdf]

# *Supplementary Material*

The following Supporting Information is available for this article:

**Table S1:** Drought dataset (separate excel file)

**Table S2:** Control dataset (separate excel file)

**Methods S1:** Connection to canopy conductance

**Methods S2:** Numerical optimization routines

**Methods S3:** Implications for photosynthesis and carbon uptake

**Figure S1:** Leave-One-Out clustering examples

**Methods S1:** Connection to canopy conductance

Under steady-state conditions, the imposed transpiration flux balances the sapwood flux induced by the forcing pressure $\Delta\psi(t)$ (McDowell et al., 2016; Whitehead, Edwards, & Jarvis, 1984).

Alongside the vapour pressure deficit and leaf conductivity, $\Delta\psi$ is a main driver of transpiration, and is thus coupled to canopy conductance. The forcing pressure $\Delta\psi$ and canopy conductivity can be linked by Darcy's law (McDowell et al., 2016). Adopting the mathematical definitions and nomenclature of Martínez-Vilalta *et al.*, (2014), we solve the canopy conductance $g_C$ as follows:

$$\boldsymbol{g_C(t)} = \frac{\boldsymbol{k_s\, A_s \Delta\psi(t)}}{\boldsymbol{A_L\, VPD}}, \tag{S1}$$

where $A_s$and $A_L$ are the sapwood and leaf areas, respectively, $k_s$ is the sapwood conductivity, and $VPD$ denotes the vapour pressure deficit (all variables and their units are listed in Table 2).

As the leaf water potential and stomatal conductance are usually mutually dependent, $\psi_L$ and $\mathrm{g_C}$ are also interdependent. Here we simplify Eq. (S1) such that $g_C$ depends on $\psi_L$ alone, and $\psi_L$ is independent of $g_C$. Over the time scale of the $g_C$ calculation (daily in this case), we assume that $A_s$ and $A_L$ are constant for a given plant canopy, meaning that $k_s$ depends on $\psi_L$ only through the possible induction of cavitation. Under this condition, the $g_C$ dynamics of Eq. (S1) is a function of $\psi_L, \psi_s$ and $VPD$ alone.

**Methods S2:** Numerical optimization routines

We used three different heuristic minimization techniques: “random search”, “simulated annealing” and “differential evolution” to ensure that minimum parameter set found by the algorithms was global across the selected parameter domain. All minimization routines were translated into / implemented in CPP. The method “random search” method was implemented based on Archetti & Schoen, (1984) with fixed step size, the “differential evolution” implementation was based on Price, Storn, & Lampinen, (2005) and the “simulated annealing” method on Caires, Italiano, Monteiro, Palamidessi, & Yung, (2005).

Applying all three techniques to our dataset always results in an equal minimum parameter set (within a 5 decimal places). To further ensure quality of the minimization techniques, each algorithm was tested extensively with the test suite of Surjanovic, S; Bingham (2013).

We selected an implicit-midpoint-ordinary-differential-equation-solver for equation 8, because it combined system stiffness robustness with performance. Using a simple ‘explicit-Euler’ solver for equation 8 exhibited numerical instability for rapid changes in soil water potential.

**Methods S3:** Implications for photosynthesis and carbon uptake

These boundary cases are defined as follows (Roman et al., 2015).

- Case 1: Extreme isohydric behavior: the change of $\psi_L(t)$ with respect to $\psi_s(t)$ is zero $\left(\frac{d\psi_L}{d\psi_s} = 0\right)$. In this case, $\psi_L$ is constant and $\Delta\psi$ decreases as the soil dries. By Eq. (S1), a decrease in $\Delta\psi$ implies a decrease in $g_C$, reduced transpiration, and consequent reduction in photosynthesis and carbon uptake.
- Case 2: Perfect isohydrodynamic behavior: the change of $\psi_L$ with respect to $\psi_s$ is equal to one $\left(\frac{d\psi_L}{d\psi_s} = 1\right)$. In this case, $\psi_L$ adjusts to maintain a constant $\Delta\psi$ as the soil dehydrates. Keeping $\Delta\psi$ constant requires maintaining a high $g_C$, ensuring that transpiration and photosynthesis continue under increasingly dry conditions. However, xylem cavitation under severe soil-moisture stress can decrease the $g_C$, thus lowering the xylem conductivity $k_s$.
- Case 3: Anisohydric behavior (Roman et al., 2015): the change of $\psi_L$ with respect to $\psi_s$ exceeds unity $\left(\frac{d\psi_L}{d\psi_s} > 1\right)$. Under drought stress, anisohydric plants adjust their $\psi_L$ until $\Delta\psi$ actually increases. Plants adopting this strategy maintain a high $g_C$ and high transpiration- and photosynthesis rates, even under strong drought stress. Loss of xylem conductivity $k_s$ induced by cavitation is compensated by the decrease in $\psi_L$ and increase in $\Delta\psi$.

Note that the classical isohydricity concept assumes an interdependence between the stomatal behavior and water potential regulation. We provide a possibility to connect leaf water potential regulation to the stomatal conductivity in Eq. (S1).

**Figure S1:**

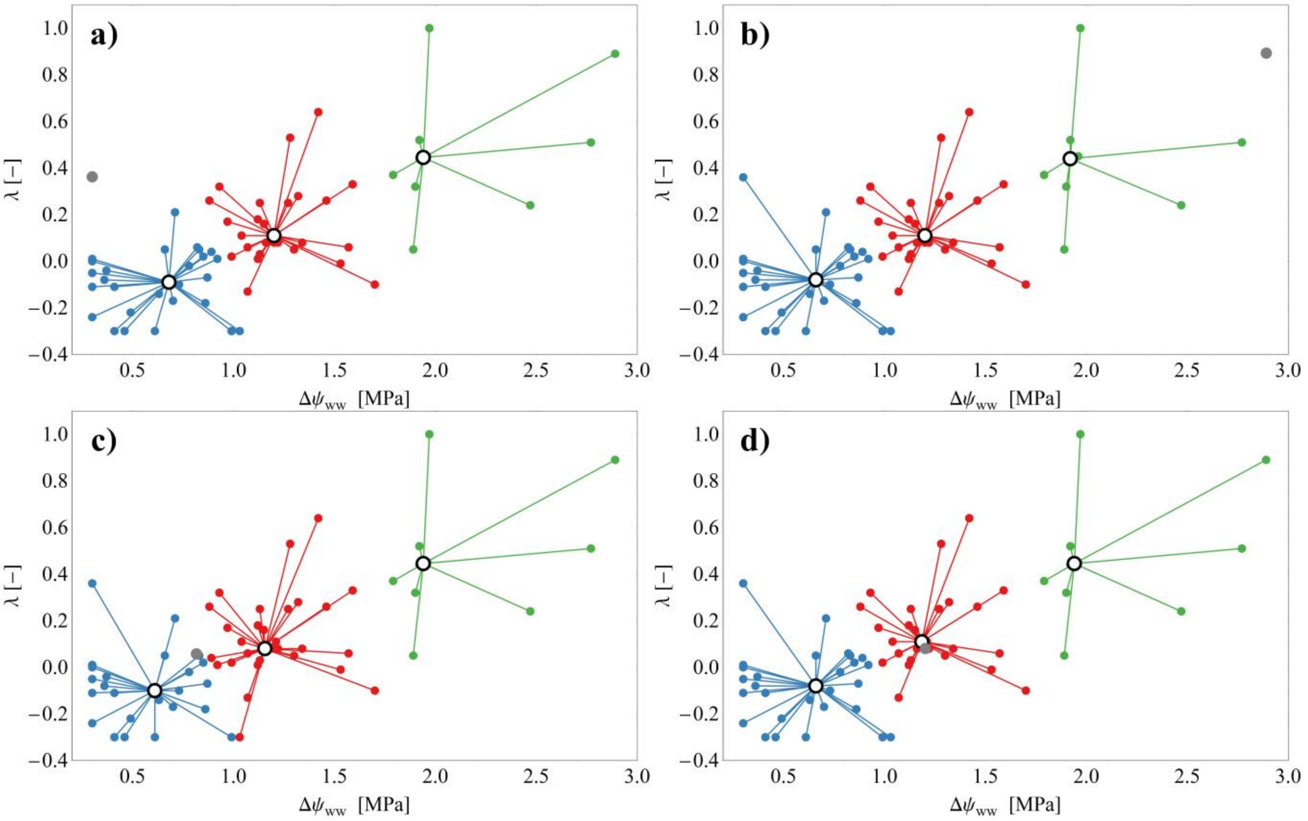


KMeans clustering based on the Leave-One-Out approach. Blue, red and green points belong to the clustering groups A, B and C, respectively. The gray point is the test dataset which reduces to one single point according to the Leave-One-Out approach. Open circles are the centroids of each of the three clusters. a) Emerging clusters leaving out the 45$^{th}$ entry (gray point) of the drought dataset: (0.3 MPa, 0.36). b) Emerging clusters leaving out the 64$^{th}$ entry (gray point) of the drought dataset: (2.9 MPa, 0.8). c) Emerging clusters leaving out the 8$^{th}$ entry (gray point) of the drought dataset: (0.8 MPa, 0.06). d) Emerging clusters leaving out the 16$^{th}$ entry (gray point) of the drought dataset: (1.2 MPa, 0.11)

**References**


Archetti, F., & Schoen, F. (1984). A survey on the global optimization problem: General theory and computational approaches. *Annals of Operations Research*, *1*(2), 87–110. https://doi.org/10.1007/BF01876141

Caires, L., Italiano, G. F., Monteiro, L., Palamidessi, C., & Yung, M. (2005). *Automata, Languages and Programming* (L. Caires, G. F. Italiano, L. Monteiro, C. Palamidessi, & M. Yung, eds.). https://doi.org/10.1007/11523468

Martínez-Vilalta, J., Poyatos, R., Aguadé, D., Retana, J., & Mencuccini, M. (2014). A new look at water transport regulation in plants. *New Phytologist*, *204*(1), 105–115. https://doi.org/10.1111/nph.12912

McDowell, N. G., Williams, A. P., Xu, C., Pockman, W. T., Dickman, L. T., Sevanto, S., … Koven, C. (2016). Multi-scale predictions of massive conifer mortality due to chronic temperature rise. *Nature Climate Change*, *6*(3), 295–300. https://doi.org/10.1038/nclimate2873

Price, K. V., Storn, R. M., & Lampinen, J. A. (Eds.). (2005). *Differential Evolution - A Practical Approach to Global Optimization*. https://doi.org/10.1007/3-540-31306-0

Roman, D. T., Novick, K. A., Brzostek, E. R., Dragoni, D., Rahman, F., & Phillips, R. P. (2015). The role of isohydric and anisohydric species in determining ecosystem-scale response to severe drought. *Oecologia*, *179*(3), 641–654. https://doi.org/10.1007/s00442-015-3380-9

Surjanovic, S; Bingham, D. (2013). Virtual Library of Simulation Experiments: Test Functions and Datasets. Retrieved from http://www.sfu.ca/~ssurjano

Whitehead, D., Edwards, W. R. N., & Jarvis, P. G. (1984). Conducting sapwood area, foliage area, and permeability in mature trees of Picea sitchensis and Pinus contorta. *Canadian Journal of Forest Research*, *14*(6), 940–947. https://doi.org/10.1139/x84-166
